# Effects of dual-task and walking speed on gait variability in people with chronic ankle instability: a cross-sectional study

**DOI:** 10.1186/s12891-017-1675-1

**Published:** 2017-07-21

**Authors:** Shmuel Springer, Uri Gottlieb

**Affiliations:** 1 Faculty of Health Science, Department of Physical Therapy, Ariel University, Ariel, Israel; 2 Israel Defense Force Medical Corps, Zerifin, Israel

**Keywords:** Ankle sprain, Chronic ankle instability, Walking, Gait variability

## Abstract

**Background:**

Recent evidence suggests that impaired central sensorimotor integration may contribute to deficits in movement control experienced by people with chronic ankle instability (CAI). This study compared the effects of dual-task and walking speed on gait variability in individuals with and without CAI.

**Methods:**

Sixteen subjects with CAI and 16 age- and gender-matched, able-bodied controls participated in this study. Stride time variability and stride length variability were measured on a treadmill under four different conditions: self-paced walking, self-paced walking with dual-task, fast walking, and fast walking with dual-task.

**Results:**

Under self-paced walking (without dual-task) there was no difference in stride time variability between CAI and control groups (*P* = 0.346). In the control group, compared to self-paced walking, stride time variability decreased in all conditions: self-paced walking with dual-task, fast speed, and fast speed with dual-task (*P* = 0.011, *P* = 0.016, *P* = 0.001, respectively). However, in the CAI group, compared to self-paced walking, decreased stride time variability was demonstrated only in the fast speed with dual-task condition (*P* = 1.000, *P* = 0.471, *P* = 0.008; respectively). Stride length variability did not change under any condition in either group.

**Conclusions:**

Subjects with CAI and healthy controls reduced their stride time variability in response to challenging walking conditions; however, the pattern of change was different. A higher level of gait disturbance was required to cause a change in walking in the CAI group compared to healthy individuals, which may indicate lower adaptability of the sensorimotor system. Clinicians may use this information and employ activities to enhance sensorimotor control during gait, when designing intervention programs for people with CAI.

The study was registered with the Clinical Trials network (registration NCT02745834, registration date 15/3/2016).

## Background

Recurrent ankle sprains occur in up to 40% of individuals who have previously experienced a lateral ankle sprain [1, 2]. Individuals who report residual symptoms, which include repetitive episodes of ‘giving way’ and subjective feeling of ankle joint instability are termed as having chronic ankle instability (CAI) [3]. The cause of these symptoms and the high frequency of recurrent ankle sprain is not fully understood [4]. It has been suggested that the residual joint instability and the high reoccurrence rates can be attributed to loss of sensory input from articular mechano-receptors, decreased muscle strength, mechanical instability of the ankle joint, and reduced ankle range of motion [5, 6].

Recent evidence suggests that deficits in central neural sensorimotor integration can contribute to impaired movement control in people with CAI [7–14]. For example, Springer et al. [8] assessed the correlation between single-limb stance postural control (Overall Stability Index) and shoulder position sense (Absolute Error Score) among people with CAI and healthy controls. Correlations between the lower and upper limbs were observed only in the healthy controls, indicating altered sensorimotor integration in the CAI group. Several studies have observed altered gait mechanism in people with CAI, which was explained by compromised central nervous system (CNS) control [9, 14–16]. It was shown that people with CAI have a typical gait pattern of increased inversion kinematics and kinetics, lateral shift of body weight, increased hip flexion during terminal swing to mid stance, reduced hip extension and increased knee flexion during terminal stance to initial swing, and slow weight transfer at the beginning and end of the stance [15–17]. Altered biomechanical strategies during gait initiation and termination tasks (e.g., reduced center of pressure displacement), have also been demonstrated in this population [9, 14]. Studies that assessed movement variability, such as knee and hip joint motions during single leg jump landing, identified differences between individuals with and without CAI, which may also indicate central motor programming deficits [10–13]. Hence, further investigation of motor control adaptations may contribute to understanding the underlying neurophysiologic mechanisms of CAI.

Gait speed and other spatio-temporal parameters during daily activities should reflect behavioral goals and environmental conditions [18]. Studies revealed that walking speed has a significant effect on joint coordination pattern and gait variability [18–20]. Therefore, assessing gait variability under challenging situations such as walking at different speeds might test CNS flexibility in controlling gait [19, 20]. Moreover, based on the understanding that for many daily activities even a fully intact motor control system requires attention and cognitive resources [21], the dual-task paradigm has been used to provide insight into the demands of postural control and gait on attention. Performance of a cognitive task has been shown to decrease postural control in participants with CAI as compared to healthy controls [7, 22]. However, no previous study examined the impact of cognitive task and walking speed on gait performance in subjects with CAI.

Balance during walking is reflected by precise spatial and temporal control of foot placement. Stride to stride fluctuations in time and length are related to control of the rhythmic walking mechanism. Thus, previous research has suggested that studying gait variability is a reliable way to quantify locomotion [23]. The mechanism of adjusting movement variability is considered beneficial for coping with changes, maintaining stability, preventing injury, and attaining higher motor skills [24]. Performing a cognitive task while walking or while altering self-paced walking speed has been related to changes in gait variability in populations with neurological and musculoskeletal pathologies, as well in healthy young individuals [25–28]. Yet, there is no consensus in the literature as to how to interpret these changes. Decreased variability while performing demanding gait tasks may reflect voluntary gait adaptation toward a more conservative gait pattern [26]. Alternatively, it has been suggested that increased variability may indicate CNS flexibility and adaptability to changes in task demands [29]. A possible central sensorimotor control deficit in people with CAI may constrain the ability of the CNS to adjust to different task demands; thus, affecting central control over gait variability and reducing the ability to cope with varied tasks. Consequently, testing the mechanism of adjusting gait variability as a response to complex walking conditions in people with CAI compared to healthy controls may provide more information on sensorimotor control in this population.

The present study was designed to compare the effects of dual-task and walking speed on gait variability in individuals with and without CAI. Previous reports, including a meta-analysis, indicated that simple postural tasks do not always discriminate between participants with CAI and those without [6, 8, 30]. Consequently, we hypothesized that gait variability among individuals with and without CAI will be similar during “normal” self-paced walking, whereas gait will vary under complex walking conditions.

## Methods

### Participants

Sixteen participants with CAI and 16 age- and gender-matched, able-bodied controls volunteered to participate in the study. All participants were recruited from military clinics. The enrollment criteria for the CAI group were based on inclusion criteria for investigating CAI as suggested by Delahunt et al. [3] and the position statement of the International Ankle Consortium [31–33]. Participants were recruited for the CAI group if they met the following criteria: (i) history of at least one significant ankle sprain which occurred at least 12 months prior to enrollment in the study and was diagnosed by a physician or a physical therapist using clinical examination classifications described by Malliaropoulos et al. [34], (ii) a history of at least two episodes of ‘giving way’, and feelings of ankle joint instability in the previously injured ankle joint [3], (iii) the most recent injury occurred more than 6 weeks prior to study enrollment, (iv) a positive response to at least five yes/no questions (question 1, plus four others) of the Ankle Instability Instrument [35], and (v) able to bear full weight on the injured lower extremity with no more than mild discomfort. Exclusion criteria for this group were evidence of concomitant injury (such as bony injury or significant muscular/tendon injury), previous ankle surgery, other pathological conditions or surgical procedures in the lower extremity, or neurological/vestibular or any other balance disorder.

The control group included healthy participants. Enrollment criteria were no history of ankle sprains, no current or previous conditions that could affect gait or balance (in particular ankle joint injury within the past 6 months), chronic disease (e.g., multiple sclerosis, stroke, Parkinson’s disease, rheumatoid arthritis, or type 2 diabetes), or history of visual or vestibular disturbance affecting balance.

The study was approved by the Israel Defense Force Medical Corps Ethical Review Board (approval number IDF-1482-2014). All participants provided written informed consent. The study was registered with the Clinical Trials network (registration NCT02745834).

### Procedure

Gait was evaluated while the subjects walked on a treadmill under four different walking conditions: self-paced walking speed, self-paced walking with dual-task, fast walking, and fast walking with dual-task. Under the self-paced walking condition, subjects were instructed to walk at their normal self-selected pace, whereas under the fast condition the instruction was to “walk as quickly as possible without running.” In order to set walking speed, gait conditions without dual-task were measured before the same condition with dual-task, while the order of self-paced/fast walking was randomized.

Stride time variability (STV) [100 X (standard deviation of stride time/mean stride time)] and stride length variability (SLV) [100 X (standard deviation of stride length/mean stride length)] were measured using the OPTOGait system (Microgate, Bolzano, Italy) [36]. The OPTOGait system consists of a transmitter and receiver bars, each 1 m long, located on both sides of the treadmill. The transmitter bar has 99 infrared LEDs and the receiver bar has 99 sensors. Stepping between the bars blocks the infrared rays, allowing the system to obtain spatio-temporal gait parameters without the use of additional markers. Data were sampled at 1000 Hz and processed using dedicated software (Optojump Next, Version 1.3.20.0, Microgate, Bolzano, Italy) [36]. Subjects performed one, 2-min trial, of each of the 4 walking conditions, with 2 min rest between tests. In addition, subjects were provided with an opportunity for conditioning by taking several steps on the treadmill before data were collected. During the dual-task conditions, subjects walked while reciting out loud serial subtractions of 7, starting from a different 3 digit number at each trial. No instructions regarding priority of walking vs. cognitive task were given. Before performing the task while walking, the arithmetic task was measured for 120 s while sitting, to examine the effects of walking on this cognitive task. To assess the dual-task performance, a normalized response index was calculated based on number of correct responses/number of total responses × 14, as described by Hayman [37].

### Statistical analysis

Descriptive statistics included means and standard deviations (M ± SD). A t-test was used to compare baseline characteristics (age, gender, height, weight, and self-paced/fast gait speed) between the CAI and control groups. Two separate 2 × 2× 2 (group × task × speed) mixed model ANOVAs were performed to examine the effect of group, and 2 within subject factors (dual-task and gait speed) on STV and SLV. Analyses of variance were followed by post hoc analyses with Bonferroni corrections, as appropriate. Additional 2× 3 (group × testing condition) mixed model ANOVA was done to examine the effects of group and testing conditions as repeated measures (sitting, normal walking, and fast walking) on the serial-7 task performance. Significance was determined at *P* < 0.05. The analysis was conducted using IBM SPSS V22 (SPSS, Inc., Chicago, Illinois).

## Results

### Subject characteristics

Subject characteristics are summarized in Table 1. There were no differences in baseline characteristics (age, gender, height, weight, and self-paced/fast gait speed) between groups. The average time since last sprain in the CAI group was 21.25 ± 16.57 weeks and the average Ankle Instability Instrument score was 6.81 ± 1.38.

**Table 1:** Subject characteristics

Parameter	Group		*P*-value
	CAI (*n* = 16)	Control (*n* = 16)	
Age (years) mean ± SD	20.97 ± 4.19	21.41 ± 4.94	0.521
Height (cm) mean ± SD	170.12 ± 6.66	169.25 ± 9.29	0.215
Weight (kg) mean ± SD	65.44 ± 9.77	62.00 ± 11.38	0.804
Gender (F/M)	9/16	9/16	---
SP speed (m/s) mean ± SD	1.32 ± 0.15	1.38 ± 0.18	0.288
Fast speed (m/s) mean ± SD	1.70 ± 0.19	1.65 ± 0.17	0.457
Ankle with recurrent sprains (RT/LT/BIL)	3/6/7	---	---
Time since last sprain (weeks) mean ± SD	21.25 ± 16.57	---	---
Ankle Instability Instrument score	6.81 ± 1.38	---	---

*CAI* chronic ankle instability, *SP* self-paced, *RT* right, *LT* left, *BIL* bilateral

### Serial-7 task performance

Table 2 summarizes performance in the serial-7 subtraction task. ANOVA showed no significant effects of group or testing condition, indicating that the serial-7 performance was similar between groups and during sitting and self-paced/fast dual-task walking conditions.

**Table 2:** Serial-7 performance

	Sitting		Self-paced walking		Fast walking	
	CAI	Control	CAI	Control	CAI	Control
Attempts	24.94 ± 14.28	23.19 ± 9.79	26.88 ± 11.46	27.38 ± 12.82	27.13 ± 11.79	25.31 ± 13.51
Correct	23.31 ± 14.75	19.75 ± 11.76	25.50 ± 11.71	23.63 ± 12.84	25.38 ± 12.37	23.19 ± 13.60
Error	1.63 ± 1.54	3.44 ± 3.44	1.38 ± 1.45	3.69 ± 4.95	1.75 ± 1.84	2.13 ± 2.36
Response Index	12.80 ± 1.12	11.15 ± 3.55	13.07 ± 1.12	11.74 ± 2.28	12.82 ± 1.41	12.37 ± 1.95

### Gait variability

**Fig. 1:**
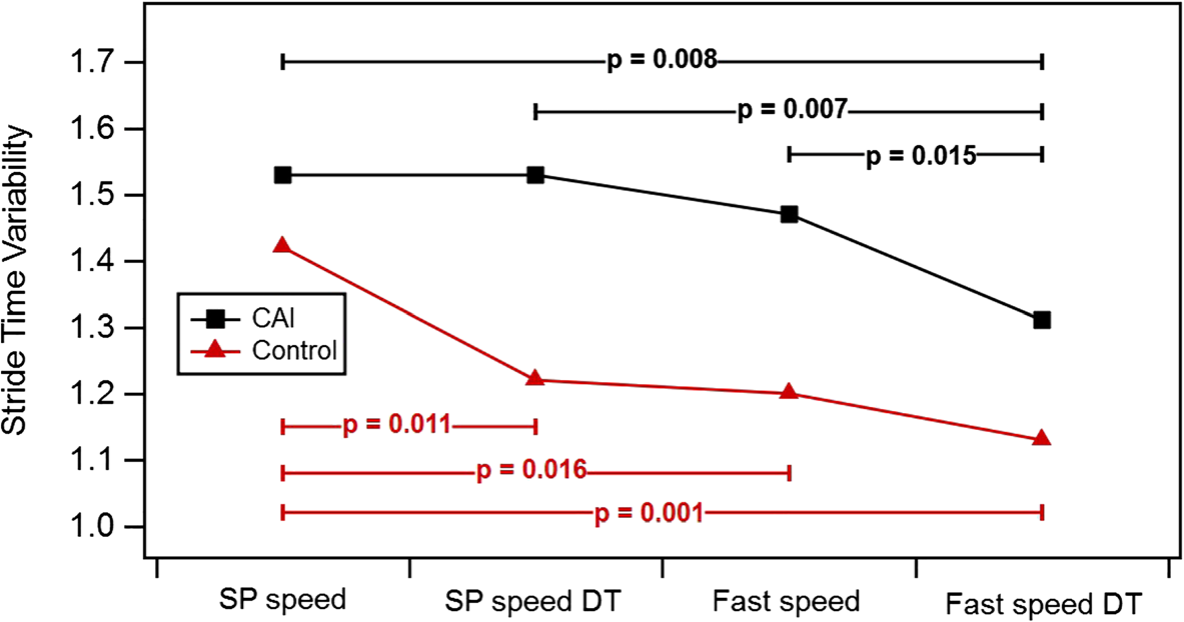
Stride time variability results of the two groups under all gait conditions. CAI- chronic ankle instability, SP- self-paced, DT- dual task

Table 3 presents the means and standard deviations of the gait variability outcomes (STV/SLV) under each walking condition in both groups, as well as post hoc comparisons between groups, where appropriate. STV results of the two groups under all 4 gait conditions are also presented in Fig. 1. The ANOVA that examined the effects of group, dual-task, and gait speed on SLV indicated that none of these 3 parameters had a significant effect.

**Table 3:** Means ± standard deviations of gait variability outcomes under each walking condition in both groups, and post hoc comparisons between groups@^a^

Group	Stride time variability (%)			
	SP speed	SP speed DT	Fast speed	Fast speed DT
CAI	1.53 ± 0.35	1.53 ± 0.43	1.47 ± 0.34	1.31 ± 0.32
Control (*n* = 16)	1.42 ± 0.32	1.22 ± 0.26	1.20 ± 0.29	1.13 ± 0.24
Between group comparisons	0.346	0.019	0.024	0.079
Group	Stride length variability (%)			
	SP speed	SP speed DT	Fast speed	Fast speed DT
CAI	1.57 ± 0.38	1.53 ± 0.41	1.44 ± 0.35	1.45 ± 0.65
Control (*n* = 16)	1.43 ± 0.39	1.18 ± 0.40	1.18 ± 0.26	1.15 ± 0.42

@^a^As the ANOVA that examined the effect of group, dual-task, and gait speed on stride length variability had no significant effect, no post hoc comparisons were made

*CAI* chronic ankle instability, *DT* dual-task, *SP* self-paced

The ANOVA that examined the effect of group, dual-task, and gait speed on STV yielded significant effects for all 3 parameters (*P* = 0.022, *P* = 0.014, and *P* = 0.008, respectively). An interaction effect was also found for group × dual-task × speed (*P* = 0.007).

Under the self-paced walking condition without dual-task, there was no difference in STV between groups (*P* = 0.346; Table 3).

In the control group, compared to self-paced walking, STV decreased in all conditions: self-paced walking with dual-task, fast speed, and fast speed with dual-task (*P* = 0.011, *P* = 0.016, and *P* = 0.001, respectively). However, in the CAI group, compared to self-paced walking, STV was decreased only in the fast speed with dual-task condition (*P* = 1.000, *P* = 0.471, and *P* = 0.008, respectively).

Additional comparisons indicated decreased STV in the CAI group when the fast speed dual-task condition was compared to fast speed (*P* = 0.0015), and self-paced speed with dual-task (*P* = 0.007). No change was observed in the control group (*P* = 0.264 and *P* = 0.256, respectively).

## Discussion

The results of the present study show that subjects with CAI and healthy controls reduced their STV in response to challenging walking conditions, although the pattern of change differed. In the healthy control group, decreased STV was observed when the normal self-paced walking speed condition was compared to the more challenging conditions such as self-paced speed with dual-task or fast speed; however, additional complexity in walking, such as fast walking with dual-task did not further change gait rhythmicity. In contrast, among subjects with CAI the degree of STV in normal self-paced speed was maintained in self-paced speed with dual-task and the fast speed conditions; yet, a significant decrease in STV was demonstrated in fast walking with dual-task. As gait variability reflects CNS control of walking [23, 29], these findings could indicate differing CNS adaptability patterns in the two groups. It seems that a higher level of gait disturbance is required to cause reduced STV in the CAI group compared to healthy individuals. The present investigation extends the growing body of evidence suggesting that altered movement control in people with CAI may be attributed to CNS deficits [7–9].

It was suggested that reduced STV during complex walking conditions reflects a reorganization of the sensorimotor system toward a more stable pattern of movement execution [38]. This coping mechanism of adjusting variability may assist in preventing injury during complex walking tasks [24]. The higher degree of gait disturbance required to initiate STV change in the CAI group could indicate CNS difficulty in reorganizing the movement pattern. While healthy subjects might react to slight changes in task and environmental circumstances, subjects with CAI might have delayed or insufficient reactions, which might impair their ability to balance gait and could explain the tendency towards repeated ankle sprains, as well as associated recurring symptoms.

Our findings are consistent with previous studies, which demonstrated that individuals with CAI have reduced ability to cope spontaneously with changes in task and environment due to constraints in the sensorimotor system [30, 39]. For example, McKeon et al. [40] tested postural control during single limb stance with eyes open and eyes closed, in people with and without CAI. Differences between groups manifested only when vision was removed. The authors suggested that the behavior observed in the CAI group could arise from the unique interactions between an impaired sensorimotor system and the task of maintaining single limb stance in the absence of visual input.

It has been demonstrated that altered gait mechanics are associated with CAI and that ankle sprains commonly occur during gait [9, 14–16]. Based on our results, clinicians may want to consider training subjects with CAI under varied gait conditions, such as walking at different speeds and with dual-task, in order to prevent recurrent injury. This practice may improve the ability of the sensorimotor system to reorganize movement and to adapt to varied conditions. Nevertheless, it should be emphasized that while balance training programs have been shown to improve function in people with CAI [13, 39], evidence to support training to improve functional activities, such as gait performance, is currently lacking [41]. Thus, further investigations should be performed to test the efficacy of such training.

While the pattern of decreased STV during the fast and dual-task walking conditions differed between groups, STV during self-paced walking did not. Similarly, recent systematic reviews [6, 30] indicated that simple postural stability tests such as single-leg stance may not always discriminate between individuals with CAI and healthy controls. Therefore, assessments with more challenging tasks, such as dual-task or fast walking, and evaluation of gait kinematics and kinetics may be more appropriate for testing impairments related to CAI.

The similarity of the tests in the present study to normal function enhances their ecological validity. Nevertheless, it should be noted that while gait assessments may replicate normal function, they do not provoke movement patterns that occur during sports and other high-risk activities (e.g., landing from a jump). A comprehensive assessment should include such functional tasks when appropriate.

The present findings of decreased STV in response to challenging walking conditions support the suggestion that in young adults, changes in task and environment might redirect gait toward a more stable, less varied mechanism. However, previous investigations reporting the effects of cognitive load and speed on gait variability in young adults had conflicting results. Several studies reported increased variability during dual-task [42, 43] or fast/slow paced gait speed [19, 44], some reported decreased variability with dual-task [26, 27] or slow gait speed [27], while others demonstrated no effect of dual-task on gait variability [45, 46]. Wrightson et al. [27] suggested that the disparities between the results could be related to the method of measuring gait and the differences between over-ground and treadmill walking. Other explanations could be related to difference in the cognitive task employed or to the instruction provided for how to decrease or increase self-paced walking speed. None of the above provides a comprehensive explanation for the diverse results. Thus, further investigation is required to fully understand the effects of changes in the task and environment on gait mechanism in young adults.

Our results indicated no effects of group or testing condition on performance in the serial-7 subtraction dual-task. This may indicate that participants in both groups prioritized the cognitive task. Previous research suggested that while simultaneously performing another task, young adults prioritized the postural assignment, known as ‘posture first’ paradigm [47]. However, a recent integrated model of task prioritization proposed by Yogev-Seligmann et al. [48] suggested that task prioritization might be related to factors such as postural reserve. In consonance with this model, it can be assumed that the participants in both groups had sufficient postural reserve (i.e., muscle strength and anticipatory mechanisms). Yet, as mentioned above, the ability to adjust dynamic posture strategy according to walking complexity differed between groups.

Although the cognitive dual-task and gait speed had a significant effect on STV, it did not influence SLV. This finding is in agreement with previous studies [42, 43] that showed that dual cognitive task provoked significant changes in STV but not in length variability among healthy adults. As suggested by Beauchet et al. [42] this may indicate that control of the spatial rhythmic stepping mechanism requires less CNS resources than does temporal control.

The present study had several limitations. One potential limitation was related to measuring self-paced gait speed and variability while subjects walked on a treadmill. Yet, it has been demonstrated that self-paced treadmill walking provides a reliable record of typical self-paced gait speed [49], and that variability measured by treadmill walking may be an acceptable representation of over-ground walking [50]. It should also be noted that as all subjects were measured under the same conditions, it could be expected that the same differences would be observed in over-ground walking. Another limitation is related to the complexity of the gait conditions. While the present investigation augmented the complexity of self-paced walking by increasing gait speed, adding a serial-7 dual-task, or both, many situations challenge the regular self-paced gait. Future investigations should examine the effect of varied gait conditions on gait performance in people with CAI. Finally, this study included young subjects with a narrow age range, as well as subjects who had bilateral and unilateral ankle sprains. CAI is common in older adults as well as in children [51] and the mixed cohort of the CAI group might have affected the results. Further studies that will test the influence of these factors are warranted.

## Conclusions

The present study demonstrated that both subjects with CAI and healthy controls reduced STV in response to challenging walking conditions. However, a more complex walking condition was required for the subjects with CAI to reduce their STV. This may be explained by a limited ability of the sensorimotor system to reorganize movement patterns under varying gait conditions. Clinicians should consider how to address this issue when designing intervention programs for people with CAI.

## Abbreviations

CAI: chronic ankle instability
CNS: central nervous system
SLV: stride length variability
STV: stride time variability
